# Supplementary material for: On predictors of misconceptions about educational topics: A case of topic specificity
Source: PLoS One. 2021 Dec 1;16(12):e0259878. doi: 10.1371/journal.pone.0259878 (PMC8635341; doi:10.1371/journal.pone.0259878)

# S1 Tables. Results including outliers.

## Participants

Initial sample size: *N* = **422**

After exclusion of non-students: *N* = **406**

After exclusion because of duration (final sample size): *N* = **369**

Mean duration time: *M* = **19.61** min. (*SD* = **29.32** min.)

## Table 1. Field of study.

| Field of study | *n* | % |
| --- | --- | --- |
| pre_taeachers | 121 | 35.69 |
| pedpsy | 144 | 42.48 |
| others | 74 | 21.83 |

## Table 2. Descriptive statistics of misconceptions and predictors.

|  | *N* | Items |  | *M* | CI | *SD* | Ω |
| --- | --- | --- | --- | --- | --- | --- | --- |
| *Misconceptions^a^* |  |  |  |  |  |  |  |
| Class size | 360 | 4 |  | 4.78 | [4.69; 4.88] | 0.92 | .77 |
| Grade retention | 357 | 7 |  | 3.38 | [3.29; 3.46] | 0.84 | .78 |
| Direct instruction | 360 | 5 |  | 4.17 | [4.08; 4.26] | 0.84 | .66 |
| Feminization | 358 | 4 |  | 1.92 | [1.83; 2.02] | 0.90 | .90 |
| *Study-related charecteristics* |  |  |  |  |  |  |  |
| Study progress^b^ | 369 | 1 |  | 4.98 | [4.63; 5.33] | 3.52 | - |
| School-based experience^c^ | 118 | 1 |  | 13.64 | [9.92; 17.36] | 20.69 | - |
| *Cognitive ability* |  |  |  |  |  |  |  |
| Numeracy^d^ | 328 | 4 |  | 1.69 | [1.55; 1.83] | 1.24 | .82 |
| *Epistemic orientations^e^* |  |  |  |  |  |  |  |
| Trust in intuition | 339 | 4 |  | 5.40 | [5.25; 5.54] | 1.36 | .76 |
| Need for evidence | 339 | 4 |  | 6.31 | [6.15; 6.48] | 1.55 | .80 |
| Truth is political | 339 | 4 |  | 4.55 | [4.37; 4.74] | 1.73 | .74 |
| *Worldviews and values* |  |  |  |  |  |  |  |
| Conservative orientation^f^ | 338 | 1 |  | 3.93 | [3.75; 4.12] | 1.70 | - |
| Educational goals^g^ |  |  |  |  |  |  |  |
| Intellectual goals | 346 | 6 |  | 3.00 | [2.95; 3.05] | 0.47 | .55 |
| Social goals | 346 | 8 |  | 3.20 | [3.15; 3.24] | 0.45 | .71 |
| Conventional goals | 346 | 3 |  | 2.92 | [2.86; 2.98] | 0.58 | .65 |

^a^ Six-point Likert scale (*do not agree at all* [1] to *fully agree* [6])

^b^ Semesters of study

^c^ Number of weeks (subsample of preservice teachers only)

^d^ Number of correct answers (0–4)

^e^ Nine-point Likert scale (*do not agree at all* [1] to *fully agree* [9])

^f^ Ten-point Likert scale (*extremely liberal* [1] to *extremely conservative* [10])

^g^ Four-point Likert scale (*less important* [1] to *very important* [4]); the number of response categories varied because we used the instruments’ original answer formats, respectively.

Ω = McDonald’s Ω reliability

## Table 3. Invariance analysis of the four factor measurement model of educational misconceptions across groups of students with different field of study (i.e., teacher education, education-related, non-educational study programs).

| Model | χ² | *df* | RMSEA | SRMR | CFI | Δχ² | Δ*df* | *p* |
| --- | --- | --- | --- | --- | --- | --- | --- | --- |
| Configural | 587.620 | 387 | .068 | 0.066 | .905 |  |  |  |
| Metric | 623.345 | 415 | .067 | 0.071 | .901 |  |  |  |
| Configural vs. metric |  |  |  |  |  | 35.725 | 28 | .150 |
| Scalar | 668.675 | 443 | .067 | 0.074 | .893 |  |  |  |
| Metric vs. scalar |  |  |  |  |  | 45.330 | 28 | .020 |

## Table 4. Goodness of fit indices for the structural equation models of misconceptions about educational topics

| Model | χ² | *df* | RMSEA | CFI | SRMR |
| --- | --- | --- | --- | --- | --- |
| Class size | 110.899 | 59 | .051 | .910 | .042 |
| Grade retention | 120.847 | 73 | .044 | .929 | .042 |
| Direct instruction | 103.152 | 59 | .047 | .902 | .044 |
| Feminization | 124.894 | 73 | .046 | .951 | .039 |

## Table 5. Standardized regression estimates from the structural equation models of misconceptions about educational topics.

|  | Class Size | | | Grade Retention | | | Direct Instruction | | | Feminization | | |
| --- | --- | --- | --- | --- | --- | --- | --- | --- | --- | --- | --- | --- |
| Predictor | β | | *p* | | β | *p* | | β | *p* | | β | *p* |
| *Study-related characteristics* | | | | | | | | | | | | |
| Field of study^a^ |  | |  | |  |  | |  |  | |  |  |
| Education-related | ***.35*** | | ***.02*** | | .01 | .93 | | **.76** | **<.001** | | .12 | .34 |
| Non-educational | ***.51*** | | ***<.01*** | | .02 | .93 | | ***.69*** | ***<.001*** | | .15 | .37 |
| Study progress | -.10 | | .09 | | *-.11* | *.10* | | .01 | .83 | | -.08 | .16 |
| *Cognitive ability* | | | | | | | | | | | | |
| Numeracy | .03 | | .64 | | .09 | .27 | | -.05 | .46 | | -.01 | .92 |
| *Epistemic Orientations* | | | | | | | | | | | | |
| Faith in intuition | -.06 | | .39 | | .01 | .95 | | .06 | .50 | | -.01 | .91 |
| Need for evidence | .08 | | .40 | | .01 | .93 | | -.04 | .72 | | .18 | .04 |
| Truth is political | .04 | | .66 | | -.16 | .12 | | .10 | .28 | | .12 | .24 |
| *Worldviews and values* | | | | | | | | | | | | |
| Conservative orientation | -.16 | | .07 | | .01 | .89 | | ***-.26*** | *.****01*** | | -.03 | .70 |
| Educational goals |  | |  | |  |  | |  |  | |  |  |
| Intellectual | .00 | | .99 | | -.04 | .09 | | .05 | .82 | | -.11 | .59 |
| Social | -.02 | | .87 | | .01 | .96 | | .09 | .48 | | -.01 | .94 |
| Conventional | .14 | | .44 | | ***.58*** | ***<.001*** | | -.11 | .52 | | -.06 | .74 |
| *R*^2^ | .10 |  | | | .18 |  | | .25 |  | | .07 |  |

Estimates standardized on outcome; boldface = *p* ≤ .05.; italic = effect as predicted.

^a^ Teacher education as reference group.

**Fig 1. Misconceptions by field of study*.*** The figure shows the means and 95% CIs of the agreement to the misconceptions across three student groups. Higher values indicate higher agreement.


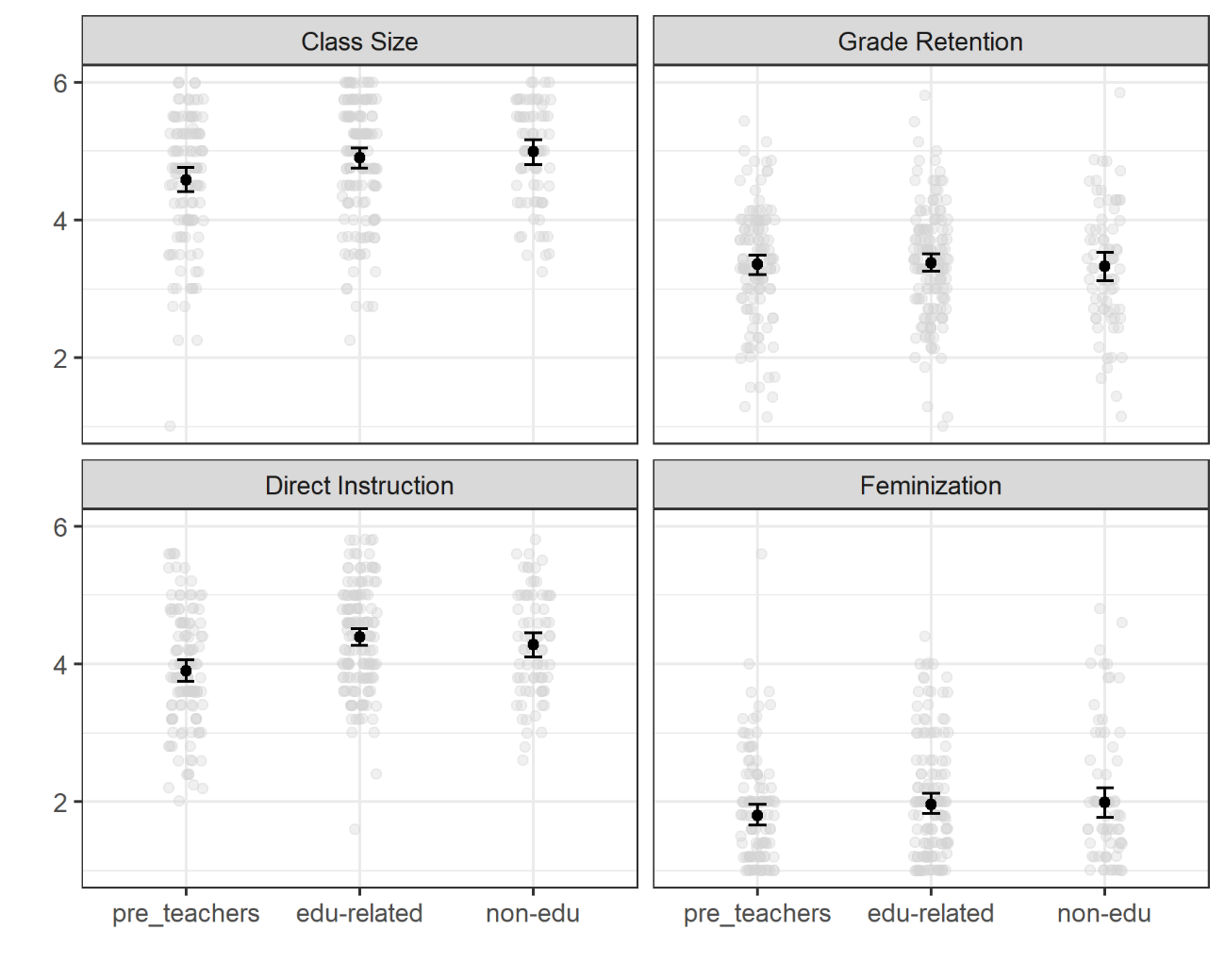

Supplement: S1 Table — (DOCX) [file pone.0259878.s001.DOCX]
