# Supplementary material for: On predictors of misconceptions about educational topics: A case of topic specificity
Source: PLoS One. 2021 Dec 1;16(12):e0259878. doi: 10.1371/journal.pone.0259878 (PMC8635341; doi:10.1371/journal.pone.0259878)
Supplement: S2 Table — (DOCX) [file pone.0259878.s002.DOCX]

# S2 Table. Results of the measurement invariance analysis of the four factor measurement model of educational misconceptions across groups of students with different field of study (i.e., teacher education, education-related, non-educational study programs).

| Model | χ² | *df* | AIC | BIC | RMSEA | SRMR | CFI | Δχ² | Δ*df* | *p* |
| --- | --- | --- | --- | --- | --- | --- | --- | --- | --- | --- |
| Configural | 492.984 | 387 | 13632 | 14289 | .054 | 0068 | .940 |  |  |  |
| Metric | 524.212 | 415 | 13607 | 14162 | .053 | .075 | .938 |  |  |  |
| Configural vs. metric |  |  |  |  |  |  |  | 31.228 | 28 | .307 |
| Scalar | 571.882 | 443 | 13599 | 14052 | .055 | .078 | 0.927 |  |  |  |
| Metric vs. scalar^a^ |  |  |  |  |  |  |  | 47.67 | 28 | .012 |

# ^a^The χ2-difference test is statistically significant, but AIC and BIC support the scalar invariance model over the metric model; ΔCFI and ΔRMSEA are small and below cut off-values by Chen (2007) and Cheung & Rensvold (2002).

# References

Chen, F. F. (2007). Sensitivity of goodness of fit indexes to lack of measurement invariance. *Structural equation modeling: a multidisciplinary journal*, *14*(3), 464-504.

Cheung, G. W., & Rensvold, R. B. (2002). Evaluating goodness-of-fit indexes for testing measurement invariance. *Structural equation modeling*, *9*(2), 233-255.
