# Supplementary material for: On predictors of misconceptions about educational topics: A case of topic specificity
Source: PLoS One. 2021 Dec 1;16(12):e0259878. doi: 10.1371/journal.pone.0259878 (PMC8635341; doi:10.1371/journal.pone.0259878)
Supplement: S3 Table — (DOCX) [file pone.0259878.s003.docx]

# S3 Table. Goodness-of-fit indices for the structural equation models.

| Model | χ² | *df* | RMSEA | CFI | SRMR |
| --- | --- | --- | --- | --- | --- |
| Class size | 88.752 | 59 | .042 | .935 | .042 |
| Grade retention | 116.617 | 73 | .046 | .928 | .044 |
| Direct instruction | 104.324 | 59 | .052 | .883 | .048 |
| Feminization | 129.859 | 73 | .053 | .941 | .041 |
