# Supplementary material for: On predictors of misconceptions about educational topics: A case of topic specificity
Source: PLoS One. 2021 Dec 1;16(12):e0259878. doi: 10.1371/journal.pone.0259878 (PMC8635341; doi:10.1371/journal.pone.0259878)
Supplement: Attachment — Submitted filename: Response to Reviewers.DOCX [file pone.0259878.s004.DOCX]

**Revisions to the article: On predictors of misconceptions about educational topics: A case of topic specificity (Manuscript Number PONE-D-21-14744)**

Dear Reviewer,

We thank the editor and the reviewer very much for their helpful comments and recommendations regarding our manuscript; we believe the manuscript has profited substantially from them.

Below, we provide a detailed account of the revisions we have made. We start with a point-by-point response to the reviewer’s comments. Next, we give a combined discussion of a concern raised by both the editor and the reviewer. For reasons of clarity, we have set the reviewer’s and editor’s original comments in bold, our responses in italics, and quotations from new or revised passages in plain text.

We hope we have addressed the reviewer’s and editor’s concerns adequately and that the reviewer judges the revised article to be suitable for publication in PLOS ONE.

~~~~~~~~~~~~~~~~~~~~~~~~~~~~~~~~~~~~~~~

REVIEWER COMMENTS

~~~~~~~~~~~~~~~~~~~~~~~~~~~~~~~~~~~~~~~

**1) I appreciated the manuscript which focused on a significant topic and appeared to be clearly organised. The sections corresponding to Introduction and Background (from Introduction to Epistemic orientations, world views, and educational values) seemed to be adequate overall.**

*Response: Thank you very much for this pleasant feedback.*

**2) Conversely, the research questions were not explicity given. Authors should explicity list them in order to favour the readers’ comprehension of the results and the conclusions. From this point of view, the sentence “The present study aimed to test the study hypotheses about the predictors of the educational misconceptions mentioned earlier” (p.10) was too vague.**

*Response: Thank you for this suggestion. We have revised the present study section and added an explicit statement of our research questions.*

Text [p. 10, l. 218]:

The goal of this study was to investigate whether and how student characteristics relate to questionable beliefs in education. In particular, we addressed the following research questions: (1) How do (i) study-related characteristics and aspects of cognitive ability, (ii) epistemic orientations, and (iii) conservative orientation and educational goals predict students’ endorsement of typical educational misconceptions? And (2) are these effects topic-specific?

In the previous section, we elaborated on the nature of potential relationships between misconceptions and the different individual factors from prior literature. Table 1 provides a summary of our assumptions. Before collecting data, we preregistered all hypotheses, study design, measures and analytic procedures (http://aspredicted.org/blind.php?x=8375mh). As aforementioned, some effects were tested exploratory when prior knowledge was too scarce to specify a clear assumption, which was a concern encountered most frequently with topic-specific variations of the effects.

**3) Moreover, the goal was not specified explicitly enough. Authors should explain why they prepared the paper and who should profit from the new knowledge acquired in their investigations. Unfortunately, authors failed to specify what type of benefits may result from this study and what type of stakeholders may be interested in it in terms of policy decisions.**

*Response: Thank you for this helpful comment. As requested, we have specified in the introduction how our findings will benefit relevant research and professional practice. We have also clarified which stakeholders will benefit from our research (i.e., researchers in the field of educational misconceptions, students and teachers in higher education, and professionals in school and other educational contexts). Because our study focuses on basic questions about factors that influence misconceptions, we are hesitant to use the present data to draw conclusions related to policy decisions.*

Text [p.5, l. 79]: This study extends prior research (both theoretically and empirically) by examining individual differences in misconceptions about educational topics. Considering the socio-scientific nature of educational topics, we specifically shed light on the relationship between misconceptions and individual epistemic orientations, worldviews, and values – factors which, despite their importance, have not been considered before. Further, this study has practical implications for the development of interventions to correct misconceptions, especially in the context of higher education. Understanding predictors of educational misconceptions can help to facilitate the teaching of research-based educational knowledge that contradicts students’ prior beliefs; this may be particularly important in teacher education.

**4) I would like to highlight the satisfactory description of the research design. Methods were adequately described.**

*Response: Thank you very much for this encouraging feedback.*

~~~~~~~~~~~~~~~~~~~~~~~~~~~~~~~~~~~~~~~

JOINT EDITOR AND REVIEWER COMMENTS

~~~~~~~~~~~~~~~~~~~~~~~~~~~~~~~~~~~~~~~

**Editor: Please could you expand on the conclusions drawn from your results to put the work into context and to explain its impact.**

**Reviewer: The results discussion had merits, but it was a bit thin in interpretations. For instance, the authors claimed: “Some of the most striking findings of this study are in fact null findings…..(p. 21, line 439). No comment? Why? Consequences?**

*Response: Thank you for this feedback. To expand the interpretations of our results, we have added two paragraphs to our discussion. First, we have added a further reason for our null findings and summarized consequences that could be drawn from them. Second, we added an “implications” section to explain the implications of our results on research and practice in higher education. At the same time, because the present study was mostly focused on basic research, we have tried to avoid speculating too much about the practical implications of our findings.*

Text [p.22, l. 480]: Third, it might be that our measures did not successfully capture the variables under investigation. Though we used measures that were applied successfully in earlier research, they may not have worked well in the context of the present study.

To summarize, misconceptions about educational topics appear persistent, and our results underline that they are also hard to predict. Moreover, as explained below, our findings have relevant theoretical and practical implications for future research and higher education.

Text [p.23, l. 487]: As previously discussed, misconceptions may occur in “clusters” that have similarities in content and structure (e.g., referring to institutional practices or teaching practices) and that maintain or reinforce each other [11]. For example, Macdonald et al. [14] identified a cluster of specific neuromyths bearing similar characteristics that could be addressed simultaneously in interventions. Future research should examine commonalities in the structure and characteristics of common topics of misconceptions. Attaining a deeper understanding of what makes an educational topic prone to misconceptions will provide an important additional perspective to address those misconceptions. If such commonalities can be identified, they may also contribute to a better understanding of the topic-specific relations found in this study and may help explain the persistence of educational misconceptions.

Furthermore, considering the socio-scientific nature of educational matters, the impact of personal views and stances needs to be considered more thoroughly. Existing intervention approaches, such as refutational texts [2,10], predominantly aim to change individuals’ beliefs by replacing dysfunctional explanations and providing accurate knowledge. However, these studies pay only little attention to individuals’ attitudes and values, which may need to be addressed in addition to cognitive factors in order to overcome misconceptions. This may be a reason why the findings on the impact of such interventions are mixed. Only recently, Thacker et al. [24] shed light on attitudes and values as influencing factors, inspecting the effects of measures they called “persuasive refutation texts”. Our results support the importance of further developing such approaches to mitigate questionable beliefs, and this may play a specific role in interventions in higher education and teacher education. In particular, preservice teachers who have been found to belief in a number of educational myths when entering teacher education programs may profit from lectures with successful debunking strategies [5]. Interventions that take into account a recipient’s personal views and stances can be promising for educating future professionals towards more evidence-based decisions and actions.

*To clarify that the paragraphs from l. 461 to l. 487 serve to explain the consequences of the null findings, we introduced these paragraphs with a question:*

Text [p.22, l. 460]: What are potential reasons for these null results?
